# Comparison of Risk of Metachronous Advanced Colorectal Neoplasia in Patients with Sporadic Adenomas Aged < 50 Versus ≥ 50 years: A Systematic Review and Meta-Analysis

**DOI:** 10.3390/jpm11020120

**Published:** 2021-02-12

**Authors:** Yoon Suk Jung, Jung Ho Park, Chan Hyuk Park

**Affiliations:** 1 Department of Internal Medicine, Kangbuk Samsung Hospital, Sungkyunkwan University School of Medicine, Seoul 03181, Korea; ys810.jung@samsung.com (Y.S.J.); jungho3.park@samsung.com (J.H.P.); 2 Department of Internal Medicine, Hanyang University Guri Hospital, Hanyang University College of Medicine, Guri 11923, Korea

**Keywords:** age, polypectomy, surveillance colonoscopy, metachronous advanced colorectal neoplasia

## Abstract

No specific recommendations are available for the surveillance of young patients aged <50 years undergoing polypectomy. We aimed to compare the risk of metachronous advanced colorectal neoplasia (ACRN) between patients aged ≥50 years and those aged <50 years who underwent polypectomy. Studies published between January 1980 and June 2020 that examined the risk of metachronous ACRN were searched. We performed a meta-analysis for the metachronous ACRN risk in patients with sporadic colorectal adenomas according to the age groups (≥50 vs. <50 years). Eight individual studies were included in the meta-analysis. The risk of metachronous ACRN was higher in patients aged ≥50 years than in those aged <50 years without significant heterogeneity (odds ratio (OR) (95% CI): 1.62 (1.34–1.96), *I*^2^ = 14%). The impact of the age group on the risk of metachronous ACRN was identified in both the low-risk (LRA) and high-risk (HRA) adenoma groups (≥50 vs. <50 years: LRA, OR 1.88 (95% CI 1.30–2.70); HRA, OR 1.50 [95% CI 1.13–2.00]). In conclusion, patients aged <50 years had a lower risk of metachronous ACRN than older patients. Young patients with sporadic adenomas do not require more intensive surveillance; rather, the surveillance interval may be extended in these patients.

## 1. Introduction

Colorectal cancer (CRC) is the second most common cause of cancer mortality worldwide [1]. CRC screening is recommended to start at the age of 50 years in adults with an average risk of CRC; accordingly, the CRC incidence has decreased in adults aged ≥50 years over the past few decades [2]. However, this trend has reversed in young adults. The incidence of CRC rises among young adults aged <50 years [2,3,4]. In keeping with these recent findings, the American Cancer Society guidelines updated in 2018 recommend that adults aged ≥45 years with an average risk of CRC undergo regular screening, either a stool-based test or a colonoscopy [5]. This is a qualified recommendation, because the simulation modeling and analysis confirmed that starting screening at age 45 years is an efficient strategy for the general population [5].

In this situation, young-onset colorectal adenoma is becoming a growing concern, and its prevalence has been reported to be increasing. In a recent meta-analysis, the overall prevalence of adenoma in young adults aged <50 years was 14.6%, based on 24 studies (n = 87,502) [6]. Two large-scale studies also showed that the prevalence of colorectal neoplasia (CRN) in adults aged 20–39 and those aged 40–49 years was 9.1% and 18.0%, respectively [7,8]. As the utilization of colonoscopy increases, the detection of sporadic adenoma among young adults is becoming more common. Patients who undergo adenoma removal have a higher incidence of subsequent adenoma than those without adenoma [9,10]. Therefore, these patients need surveillance colonoscopy after adenoma removal [9,10]. The surveillance interval is determined based on the probability of developing metachronous advanced CRN (ACRN). Metachronous lesions are lesions found on subsequent colonoscopy after polypectomy. ACRN is defined as CRC or advanced adenoma.

The current guidelines regarding post-polypectomy surveillance focus on patients aged ≥50 years and do not provide clear recommendation for the surveillance of young patients aged <50 years with adenomas [9,10]. There is marked variability in clinicians’ surveillance recommendations for young patients with sporadic adenomas [11]. Given the absence of specific recommendations for the surveillance of young-onset adenoma, these variations are not surprising. Recently, several studies have assessed the risk of metachronous ACRN in patients with young-onset adenoma. At this time, summarizing the results of these studies will help to guide clinicians in the management of young patients who underwent polypectomy. Hence, we performed a systematic review and meta-analysis for the risk of metachronous ACRN after polypectomy in patients aged <50 vs. those aged ≥50 years to secure background data that will be the basis for future guidelines on the appropriate surveillance intervals for young patients with sporadic adenomas.

## 2. Methods

### 2.1. Search Strategy

We performed a literature search for all relevant articles published between January 1980 and June 2020 that evaluated the risk of metachronous ACRN in individuals aged ≥50 and those aged <50 years using the MEDLINE, EMBASE, and Cochrane Library databases. We used the following keywords: colorectal, colorectum, colon, colonic, rectal, rectum, colonoscopy, colonoscopic, neoplasia, neoplasm, neoplasms, neoplastic, adenoma, adenomas, adenomatous, polyp, polyps, polypectomy, cancer, cancers, carcinoma, carcinomas, metachronous, index, age, and 50. The detailed search strategies are shown in Appendix A.

### 2.2. Inclusion/Exclusion Criteria

The inclusion criteria were as follows: (a) population: patients who underwent endoscopic removal of one or more sporadic adenomas, (b) intervention: aged <50 years, (c) comparator: aged ≥50 years, and (d) outcome: risk of metachronous ACRN detected during surveillance colonoscopies. Nonoriginal articles, nonhuman studies, conference abstracts, and non-English studies were excluded.

### 2.3. Study Selection

We retrieved the title and abstracts of individual studies. After removing duplicates from multiple search engines, irrelevant studies were excluded by title and abstract review. After that, we screened the full text of all remaining studies. The study selection was independently performed by two investigators (Y.S.J. and C.H.P.). Any disagreements were resolved through discussion and consensus. If agreement could not be reached, a third investigator (J.H.P) determined the final eligibility.

### 2.4. Assessment of Study Quality

Two investigators (Y.S.J. and C.H.P.) independently conducted a study quality assessment. For the observational studies, the Newcastle–Ottawa Scale was used [12]. It consists of three categories: selection (four points), comparability of the study groups (two points), and ascertainment of exposure or outcome (three points). Studies with a score of ≥7 points were considered to be of high quality. For randomized controlled trials, the Cochrane Risk of Bias assessment tool was used.

### 2.5. Data Extraction

Using a data extraction form, two investigators (Y.S.J. and C.H.P.) independently extracted the following information: first author, publication year, study design, country, study period, publication language, exclusion criteria of individual studies, and the risk of metachronous ACRN.

### 2.6. Study Endpoint

The primary endpoint of this meta-analysis was the comparative risk of metachronous ACRN between individuals with sporadic adenomas aged ≥50 years and those aged <50 years. The secondary endpoint was the pooled proportion of patients with metachronous ACRN according to the age group (≥50 vs. <50 years). We further performed meta-analyses according to the subpopulations based on the index colonoscopy findings (patients with low-risk adenoma (LRA) and those with high-risk adenoma (HRA)). Index colonoscopy was defined as an initial colonoscopy for screening. LRAs were defined as 1 to 2 tubular adenomas measuring <10mm in size, and HRAs were defined as ≥3 adenomas or advanced adenomas [9]. Advanced adenomas were defined as adenomas with size ≥10 mm, villous histology, or high-grade dysplasia [9]. ACRN included advanced adenoma and cancer [9].

### 2.7. Statistical Analysis

The meta-analysis was performed to calculate the pooled proportion of patients with metachronous ACRN according to the age group (≥50 and <50 years). For meta-analyses of comparative outcomes, the pooled odds ratios (ORs) with 95% confidence intervals (CIs) were calculated. A random-effects model was used in the meta-analyses. The heterogeneity was assessed using Cochran’s Q test, wherein *p*-values of <0.1 were considered statistically significant for heterogeneity, and *I*^2^ statistics, wherein values of >50% were suggestive of significant heterogeneity [13]. To confirm the robustness of our meta-analyses, we conducted sensitivity analyses after excluding the study that limited the upper age limit of the study participants. Funnel plot asymmetry was not evaluated when fewer than ten studies were included in the meta-analysis [14].

All *P*-values were two-tailed, and *p* < 0.05 was considered to be significant in all tests, except for heterogeneity. Analysis and reporting were performed in accordance with the Preferred Reporting Items for Systematic Reviews and Meta-Analyses guidelines [15]. All statistical procedures were conducted using the statistical software Review Manager 5.4 (version 5.4.0; Cochrane Collaboration, Copenhagen, Denmark), R (version 4.0.2; R Foundation for Statistical Computing, Vienna, Austria), and Comprehensive Meta Analysis (version 2.2.064; Biostat Inc., Englewood, NJ, USA).

## 3. Results

### 3.1. Study Selection and Characteristics

**Figure 1:**
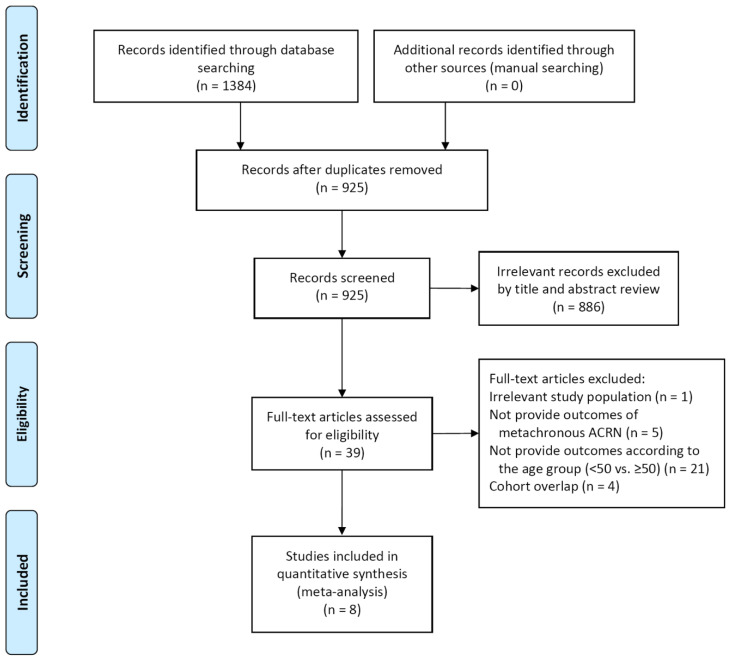
Study flow diagram. ACRN, advanced colorectal neoplasia.

Our study included eight relevant studies (Figure 1). Table 1 shows the baseline characteristics of the included studies. These studies had all been published between 2009 and 2020, with study periods ranging from 1980 to 2017 [16,17,18,19,20,21,22,23]. There were five retrospective cohort studies [17,19,20,22,23] and one case-control study [21]. The remaining two studies were a pooled analysis using patient-level data from the previous randomized controlled trials and cohort studies [16,18]. Although the study population was overlapped between these two studies, both were eligible in our study, because one reported the risk of metachronous ACRN in patients who underwent endoscopic resection of any adenomas (Martínez et al. [16]), and the other reported the risk in patients who underwent endoscopic resection of LRAs (Gupta et al. [18]). These two studies were not included together in one meta-analysis.

Among the eight studies, four were conducted in the USA [16,18,21,22], three were performed in Korea [17,19,20], and one was conducted in Israel [23]. All studies excluded patients with high-risk factors such as hereditary CRC syndrome, inflammatory bowel disease, or a history of CRC. Although the definition of metachronous ACRN slightly differed across the studies, the following definition was commonly used in most studies: “high-grade dysplasia, villous adenoma, adenoma ≥10 mm, or cancer”. In all studies except one, the ≥50 years group had no upper age limit. However, in the study by Kim, H.G., et al. [19], the ≥50 years group consisted of patients aged 50–54 years.

The quality assessment of individual studies is shown in Table 1 and Table S1. In all studies, the quality of the study was high.

### 3.2. The Proportion of Patients with Metachronous ACRN According to Age Group

**Figure 2:**
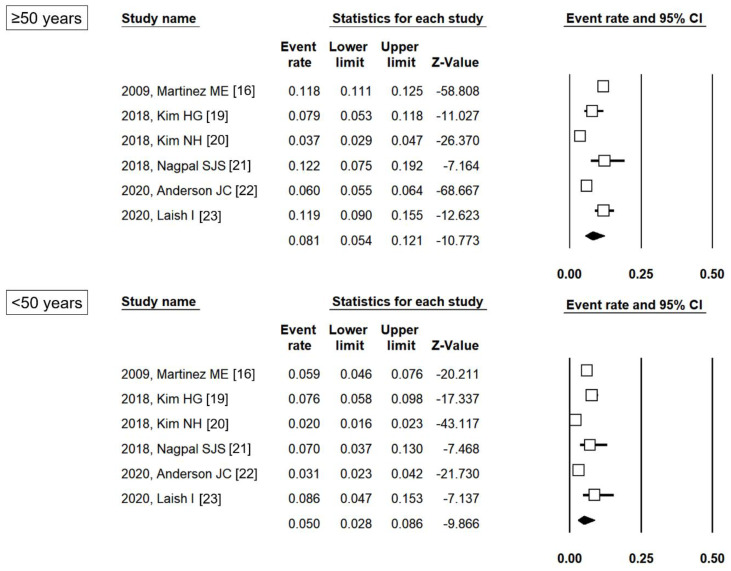
Pooled proportion of patients with metachronous ACRN according to age group. ACRN, advanced colorectal neoplasia; CI, confidence interval [16,19,20,21,22,23].

As shown in Figure 2, the pooled proportion of patients with metachronous ACRN was 8.1% (95% CI 5.4%–12.1%) and 5.0% (95% CI 2.8%–8.6%) in the ≥50 years and <50 years groups, respectively. The pooled proportion of patients with metachronous ACRN also varied according to the index colonoscopy finding (Table S2). Among patients with LRA, the pooled proportion of those with metachronous ACRN was 5.4% (95% CI 3.2%–8.8%) and 4.6% (95% CI 2.0%–10.3%) in the ≥50 years and <50 years groups, respectively, and among patients with HRA, the proportions were 10.6% (95% CI 8.5%–13.1%) and 7.3% (95% CI 5.3%–10.0%), respectively.

### 3.3. Comparative Risk of Metachronous ACRN According to Age Group

**Figure 3:**
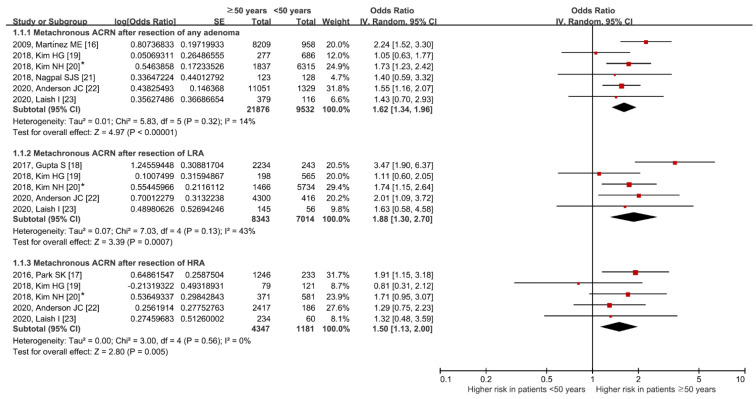
Comparative risk of metachronous ACRN between age groups according to the index colonoscopy finding groups. * Values in this study were provided by the corresponding author. ACRN, advanced colorectal neoplasia; LRA, low-risk adenoma; HRA, high-risk adenoma; SE, standard error; IV, inverse variance; CI, confidence interval [16,17,18,19,20,21,22,23].

The comparative risk of metachronous ACRN between the ≥50 years and <50 years groups is shown in Figure 3. The ≥50 years group had a higher risk of metachronous ACRN than the <50 years group (OR (95% CI): 1.62 (1.34–1.96)). No heterogeneity was identified (df = 5, *p* = 0.32, *I*^2^ = 14%).

Among patients with LRA, the risk of metachronous ACRN was higher in the ≥50 years group than in the <50 years group without significant heterogeneity (OR (95% CI): 1.88 (1.30–2.70); df = 4, *p* = 0.13, *I*^2^ = 43%). Similarly, the risk was higher in the ≥50 years group than in the <50 years group, even among patients with HRA (OR (95% CI): 1.50 (1.13–2.00); df = 4, *p* = 0.56, *I*^2^ = 0%).

### 3.4. Sensitivity Analysis

Forest plots after excluding the study by Kim, H.G., et al. [19], in which the ≥50 years group consisted of patients aged 50–54 years, are shown in Figure S1. Overall, the results of sensitivity analyses were similar to the main outcomes. Patients aged ≥50 years had a higher risk of metachronous ACRN than those aged <50 years without significant heterogeneity (OR (95% CI): 1.71 (1.43–2.05); df = 4, *p* = 0.59, *I*^2^ = 0%). The impact of age on the metachronous ACRN was also identified in both patients with LRA and HRA.

## 4. Discussion

In the present meta-analysis, we evaluated the comparative risk of metachronous ACRN after polypectomy between patients aged <50 years and ≥50 years. Patients aged <50 years had a lower risk of metachronous ACRN than those aged ≥50 years. These results were the same even if patients with LRA and those with HRA were analyzed separately.

The detection of adenoma in young individuals may lead to the diagnosis of hereditary CRC syndrome. However, the need for detecting such hereditary syndromes is extremely low among young individuals with only adenomas, although this is not uncommon in young patients with CRC (up to 16%) [24]. One study showed that of 208 young patients with adenomas, only 1 had an adenoma with mismatch repair deficiency [25]. Another study reported that, among 40 adenomas in 34 young patients, none demonstrated microsatellite instability (MSI) [26]. Even in young patients with advanced adenomas, the prevalence of MSI has been reported to be very low (5.8%, n = 3/52) [27]. In summary, most young-onset adenomas are sporadic rather than part of a hereditary syndrome. In all studies included in our meta-analysis, except for one (Anderson et al. [22]), patients with a history of CRC were excluded. Moreover, most studies excluded patients with a hereditary CRC syndrome or a strong family history of CRC (Table 1). Therefore, our results can be regarded as those for patients with sporadic adenomas.

There are no specific guidelines for the surveillance interval after polypectomy of sporadic adenomas in young patients aged <50 years. Recommendations for follow-up after polypectomy from the US Multi-Society Task Force on CRC are intended for patients aged ≥50 years [9]. It is unclear whether these recommendations are applicable for young patients aged <50 years with incidentally detected adenoma. Meanwhile, the UK guideline provides post-polypectomy surveillance recommendations according to age [10]. For high-risk patients (those with ≥2 premalignant polyps, including ≥1 advanced polyp or ≥5 premalignant polyps), the same 3-year surveillance interval is recommended for both young and old patients, whereas for low-risk patients (those with premalignant polyps but no high-risk finding), more intensive surveillance is recommended for young patients. This may be due to the concern that subsequent adenomas may develop and progress more rapidly in patients with young-onset adenomas. However, this is a weak recommendation with low-quality evidence and does not specify an absolute age standard [10].

Our meta-analysis results eliminate the concern that sporadic adenomas that develop at a younger age are more dangerous. Because the baseline adenoma characteristics (HRA vs. LRA) are strong risk factors for metachronous ACRN, we performed subgroup analysis in patients with LRA and HRA. Similar to the results of any adenoma, in both the LRA and HRA groups, patients aged <50 years had a lower risk of metachronous ACRN than those aged ≥50 years. These findings indicate that patients aged <50 years do not require closer follow-up than those aged ≥50 years, regardless of whether they have LRAs or HRAs; rather, it may be possible to extend the surveillance interval in young patients aged <50 years. However, the prerequisite here is that it is necessary to obtain a detailed family history from young patients with adenomas to confirm whether it is truly sporadic.

Given that increasing age is a strong risk factor for CRC [28], it is not surprising that younger age is associated with a lower risk of metachronous ACRN. Moreover, several studies involving patients aged <50 years have reported that older age is a risk factor for metachronous ACRN after polypectomy, although they were not included in our meta-analysis because the data in those studies were inappropriate to our meta-analysis [29,30,31,32,33]. For example, the previous two studies revealed that patients aged ≥60 years had a higher risk of metachronous ACRN than those aged <60 years [32,33]. These studies, which were not included in our meta-analysis, also support our results. Therefore, these studies and our study provide strong evidence that young patients are at a lower risk of metachronous ACRN than older patients.

We found that the pooled rates of metachronous ACRN in patients aged <50 years with any adenoma, LRA, and HRA were 5.0%, 4.6%, and 7.3%, respectively. A recent systematic review demonstrated that the pooled overall prevalence of advanced adenoma in adults aged ≥50 years was 5.2%, based on 37 studies composed of 342,121 individuals [6]. In this systematic review, the pooled rate of advanced adenoma in adults aged ≥50 years was similar to the pooled rate of metachronous ACRN in those aged <50 years in our study (5.2% vs. 5.0%). Therefore, the post-polypectomy surveillance should not be neglected in patients aged <50 years. However, it seems that there is no need to perform stronger surveillance than what is recommended by current guidelines.

This study will help to guide the surveillance interval in young patients with sporadic adenomas as the first meta-analysis comparing the risk of metachronous ACRN in patients aged ≥50 years vs. <50 years. Nevertheless, it has several limitations. First, our meta-analysis was performed based on the observational studies. Therefore, we could not determine an optimal surveillance interval in a younger population, although the comparative risk was shown between the age groups. Second, most included studies in the meta-analysis were conducted in the USA or Korea; thus, our results may not be generalized worldwide. Third, although most studies excluded patients with incomplete colonoscopy or poor bowel preparation, the adenoma detection rate (ADR), which is an important colonoscopy quality indicator, was not completely considered. Only three studies reported that the ADR met recommended thresholds [19,20,23], and only one study adjusted the ADR in multivariable analysis [22].

Despite these limitations, our meta-analysis demonstrated the risk of metachronous ACRN in young patients with sporadic adenomas. Patients with adenomas aged <50 years had a lower risk of metachronous ACRN than those aged ≥50 years. The low risk of metachronous ACRN in patients aged <50 years was also identified even after stratifying by index colonoscopy finding (low-risk vs. high-risk adenomas). These findings suggest that there is no need for young patients to have more intensive surveillance than what is recommended by current guidelines. Rather, it may be possible to extend the surveillance interval in young patients. However, these suggestions should be strictly applied only to patients with sporadic adenomas after thoroughly excluding patients with hereditary syndrome. In the future, long-term prospective studies and cost-effectiveness analyses are warranted to determine the appropriate surveillance interval for young patients with adenoma.

## Figures and Tables

**Table 1:** Baseline characteristics of the included studies and risk of metachronous lesions according to age group.

Publication Year, FIRST Author	Study Design	Study Period	Country	Exclusion Criteria	Number of Patients	Male, %	Follow-Up Duration, Years	Quality Assessment (Newcastle–Ottawa Scale: Selection/Comparability/Outcome)	Metachronous ACRN	
									Definition of Metachronous ACRN	Event and Number of Patients
2009, Martínez ME [16]	Pooled analysis using patient-level data from 7 randomized controlled trials and 1 cohort study	1980–1999 (enrollment period)	USA	Most individual studies excluded the high-risk population for CRC such as hereditary CRC syndrome *	9167	71.2	Median 47.2 (IQR 6.1–91.4)	All individual studies were assessed as having a high quality *	High-grade dysplasia, villous adenoma, or adenoma ≥ 10 mm	<50 years: 57/958 ≥50 years: 967/8209
2016, Park SK [17]	Retrospective, multicenter cohort	2004–2006 (enrollment period)	Korea	Polyposis syndrome, a history of CRC, surgical resection of the intestine, IBD, incomplete procedures	1479	73.8	<50 years: mean 4.1 (SD 1.4) 50–70 years: mean 4.0 (SD 1.4) ≥70 years: mean 4.0 (SD 1.5)	4/1/3	High-grade dysplasia, villous adenoma, adenoma ≥ 10 mm, or cancer	<50 years: 18/233 ≥50 years: 172/1246
2017, Gupta S [18]	Pooled analysis using patient-level data from six randomized controlled trials and one cohort study	1984–1999 (enrollment period)	USA	Most individual studies excluded the high-risk population for CRC, such as hereditary CRC syndrome *	2477	73.7	N/A	All individual studies were assessed as having a high quality *	High-grade dysplasia, villous adenoma, or adenoma ≥ 10 mm	<50 years: 7/243 ≥50 years: 181/2234
2018, Kim HG [19]	Retrospective, multicenter cohort	2006–2015	Korea	A strong family history of CRC, genetic syndromes, multiple (>16) adenomas at index colonoscopy, IBD, CRC, any malignancy, incomplete procedures, previous colorectal surgery	2709	59.2	N/A	4/1/3	High-grade dysplasia, villous adenoma, adenoma ≥ 10 mm, or cancer	20–49 years: 52/686 50–54 years: 22/277
2018, Kim NH [20]	Retrospective cohort	2010–2017	Korea	A history of CRC or colorectal surgery, IBD, poor bowel preparation	10014	82.9	Mean 3.4 (SD 1.4)	4/2/3	High-grade dysplasia, villous adenoma, adenoma ≥ 10 mm, or cancer	30-49 years: 124/6315 ≥50 years: 68/1837
2018, Nagpal SJS [21]	Case-control	1984–2012	USA	A personal or family history of hereditary CRC syndromes, Incomplete colonoscopy, previous colon surgery, history of CRC, IBD	251	47.4	<50 years: median 2.8 (IQR 1.3–3.9) ≥50 years: median 4.1 (IQR 2.9–5.2)	4/2/3	SSP with dysplasia, TSA, high-grade dysplasia, villous adenoma, adenoma ≥ 10 mm, or cancer	<50 years: 9/128 ≥50 years: 15/123
2020, Anderson JC [22]	Retrospective cohort	N/A	USA	Familial syndromes, IBD, incomplete colonoscopy, poor bowel preparation	12380	54.8	<40 years: mean 4.0 (SD 2.4) 40–49 years: mean 4.6 (SD 2.3) 50–59 years: mean 4.8 (SD 2.2) ≥60 years: mean 4.3 (SD 1.9)	4/2/3	High-grade dysplasia, villous adenoma, adenoma ≥ 10 mm, or cancer	<50 years: 41/1329 ≥50 years: 660/11051
2020, Laish I [23]	Retrospective cohort	2005–2015	Israel	A strong family history of CRC, hereditary syndrome, multiple (≥10) adenomas at index colonoscopy, previous colonoscopies with removal of polyps, IBD, history of CRC, previous bowel resection, incomplete procedures, ≥3 non-advanced adenomas at index colonoscopy	496	49.4	Low risk adenoma group: median 5.0 Advanced adenoma group: median 3.0	4/1/3	High-grade dysplasia, villous adenoma, adenoma ≥ 10 mm, SSP ≥ 10 mm, SSP with dysplasia, or cancer	<50 years: 10/116 ≥50 years: 45/379

* Detailed exclusion criteria and study quality assessment are demonstrated in Table S1. ACRN, advanced colorectal neoplasia; CRC, colorectal cancer; IBD, inflammatory bowel syndrome; SSP, sessile serrated polyp; TSA, traditional serrated adenoma; SD, standard deviation; IQR, interquartile range; N/A, not available.

## Data Availability

All relevant data are included in the study and supplementary information.

## Supplementary Materials

The following are available online at https://www.mdpi.com/2075-4426/11/2/120/s1, Figure S1. Sensitivity analysis after excluding the study with upper age limit. *Values in this study were provided by the corresponding author. ACRN, advanced colorectal neoplasia; LRA, low-risk adenoma; HRA, high-risk adenoma; SE, standard error; IV, inverse variance; CI, confidence interval. Table S1. Detailed exclusion criteria and quality assessment for individual studies in the two pooled analysis studies [16,18]. Table S2. Pooled proportion of patients with metachronous ACRN according to the index colonoscopy finding and age group.

## Appendix A. Detailed Search Strategy

MEDLINE (Pubmed)

(colorectal[tw] OR colorectum[tw] OR colon[tw] OR colonic[tw] OR rectal[tw] OR rectum[tw] OR colonoscopy[tw] OR colonoscopic[tw]) AND (neoplasia[tw] OR neoplasm[tw] OR neoplasms[tw] OR neoplastic[tw] OR adenoma[tw] OR adenomas[tw] OR adenomatous[tw] OR polyp[tw] OR polyps[tw] OR polypectomy[tw] OR cancer[tw] OR cancers[tw] OR carcinoma[tw] OR carcinomas[tw]) AND (metachronous[tw] OR (index colonoscopy[tw]) AND (age[tw] OR 50[title/abstract])) AND (“1980/01/01”[Date-Publication]: “3000”[Date-Publication]) NOT review[Publication Type] NOT meta-analysis[Publication Type]

EMBASE (Ovid)

1: ((colorectal or colorectum or colon or colonic or rectal or rectum or colonoscopy or colonoscopic) and (neoplasia or neoplasm or neoplasms or neoplastic or adenoma or adenomas or adenomatous or polyp or polyps or polypectomy or cancer or cancers or carcinoma or carcinomas) and (metachronous or ‘index colonoscopy’) and (age or 50)).ab,ti.

2: Limit 1 to (english language and embase and yr =“1980 -Current” and (article or article in press))

Cochrane library

#1: colorectal or colorectum or colon or colonic or rectal or rectum or colonoscopy or colonoscopic

#2: neoplasia or neoplasm or neoplasms or neoplastic or adenoma or adenomas or adenomatous or polyp or polyps or polypectomy or cancer or cancers or carcinoma or carcinomas

#3: metachronous or ‘index colonoscopy’

#4: age or 50

#5: #1 and #2 and #3 and #4 (with Cochrane Library publication date from January 1980 to 2020, in Trials).
