# Supplementary material for: Comparison of Risk of Metachronous Advanced Colorectal Neoplasia in Patients with Sporadic Adenomas Aged < 50 Versus ≥ 50 years: A Systematic Review and Meta-Analysis
Source: J Pers Med. 2021 Feb 12;11(2):120. doi: 10.3390/jpm11020120 (PMC7917624; doi:10.3390/jpm11020120)
Supplement: Supplementary file 1 [file jpm-11-00120-s001.zip › Appendix 1.docx]

**Appendix 1. Detailed search strategy**

MEDLINE (Pubmed)

(colorectal[tw] OR colorectum[tw] OR colon[tw] OR colonic[tw] OR rectal[tw] OR rectum[tw] OR colonoscopy[tw] OR colonoscopic[tw]) AND (neoplasia[tw] OR neoplasm[tw] OR neoplasms[tw] OR neoplastic[tw] OR adenoma[tw] OR adenomas[tw] OR adenomatous[tw] OR polyp[tw] OR polyps[tw] OR polypectomy[tw] OR cancer[tw] OR cancers[tw] OR carcinoma[tw] OR carcinomas[tw]) AND (metachronous[tw] OR (index colonoscopy[tw]) AND (age[tw] OR 50[title/abstract])) AND ("1980/01/01"[Date - Publication] : "3000"[Date - Publication]) NOT review[Publication Type] NOT meta-analysis[Publication Type]

EMBASE (Ovid)

1: ((colorectal or colorectum or colon or colonic or rectal or rectum or colonoscopy or colonoscopic) and (neoplasia or neoplasm or neoplasms or neoplastic or adenoma or adenomas or adenomatous or polyp or polyps or polypectomy or cancer or cancers or carcinoma or carcinomas) and (metachronous or 'index colonoscopy') and (age or 50)).ab,ti.

2: Limit 1 to (english language and embase and yr="1980 -Current" and (article or article in press))

Cochrane library

#1: colorectal or colorectum or colon or colonic or rectal or rectum or colonoscopy or colonoscopic

#2: neoplasia or neoplasm or neoplasms or neoplastic or adenoma or adenomas or adenomatous or polyp or polyps or polypectomy or cancer or cancers or carcinoma or carcinomas

#3: metachronous or 'index colonoscopy'

#4: age or 50

#5: #1 and #2 and #3 and #4 (with Cochrane Library publication date from Jan 1980 to 2020, in Trials)
