# Supplementary material for: Comparison of Risk of Metachronous Advanced Colorectal Neoplasia in Patients with Sporadic Adenomas Aged < 50 Versus ≥ 50 years: A Systematic Review and Meta-Analysis
Source: J Pers Med. 2021 Feb 12;11(2):120. doi: 10.3390/jpm11020120 (PMC7917624; doi:10.3390/jpm11020120)
Supplement: Supplementary file 1 [file jpm-11-00120-s001.zip › Supplementary Table 1.docx]

| Table S1. Detailed exclusion criteria and quality assessment for individual studies in the two pooled analysis studies [16, 18] | | | | |
| --- | --- | --- | --- | --- |
| Individual study | Pooled analysis study using patient-level data | Study design | Exclusion criteria | Quality assessment |
| Winawer *et al.*, N Engl J Med. 1993;328(13):901-6 | 2009, Martínez [16] | RCT | A family or personal history of familial polyposis, IBD, personal history of polypectomy or CRC | Low risk of bias except unclear selection bias (random sequencing generation and allocation concealment) |
| Greenberg *et al.*, N Engl J Med. 1994;331(3):141-7 | 2009, Martínez [16]  2017, Gupta [18] | RCT | Familial polyposis, invasive CRC, malabsorption syndromes | Low risk of bias except unclear selection bias (random sequencing generation and allocation concealment) |
| Baron *et al.*, N Engl J Med. 1999;340(2):101-7 | 2009, Martínez [16]  2017, Gupta [18] | RCT | Familial polyposis, invasive CRC, malabsorption syndromes | Low risk of bias except unclear selection bias (allocation concealment) |
| Alberts *et al.*, N Engl J Med. 2000;342(16):1156-62 | 2009, Martínez [16]  2017, Gupta [18] | RCT | A strong family history of CRC (≥2 FDRs with CRC), invasive cancer within the previous 5 years, a history of colon resection, severe metabolic disorders, or other severe illnesses | Low risk of bias except unclear selection bias (random sequencing generation and allocation concealment) and high risk other bias (change of allocation ratio) |
| Lieberman *et al.*, N Engl J Med. 2000;343(3):162-8 | 2009, Martínez [16]  2017, Gupta [18] | Cohort | A history of disease of the colon (colitis, polyps, or cancer) or colonic surgery, a colonic examination within the previous 10 years | Newcastle-Ottawa scale: selection 4 points / comparability 1 point / outcome 3 points |
| Schatzkin *et al.*, N Engl J Med. 2000;342(16):1149-55 | 2009, Martínez [16]  2017, Gupta [18] | RCT | Polyposis syndrome, a history of CRC, surgical resection of adenomas, bowel resection, or IBD | Low risk of bias except unclear selection bias (random sequencing generation and allocation concealment) |
| Baron *et al.,* N Engl J Med. 2003;348(10):891-9 | 2009, Martínez [16]  2017, Gupta [18] | RCT | Familial CRC syndrome, invasive CRC, malabsorption syndromes | Low risk of bias except unclear selection bias (allocation concealment) |
| Alberts *et al.*, J Natl Cancer Inst. 2005;97(11):846-53 | 2009, Martínez [16]  2017, Gupta [18] | RCT | Invasive cancer within the previous 5 years | Low risk of bias except unclear selection bias (random sequencing generation and allocation concealment) |
| The study by Martínez *et al.* investigated the risk of metachronous ACRN in patients who underwent polypectomy for any adenoma, whereas the study by Gupta *et al.* evaluated the risk of metachronous ACRN in patients with LRA at index colonoscopy. RCT, randomized controlled trial; IBD, inflammatory bowel syndrome; CRC, colorectal cancer; FDR, first-degree relative; ACRN, advanced colorectal neoplasia; LRA, low-risk adenoma | | | | |
