# Supplementary material for: Comparison of Risk of Metachronous Advanced Colorectal Neoplasia in Patients with Sporadic Adenomas Aged < 50 Versus ≥ 50 years: A Systematic Review and Meta-Analysis
Source: J Pers Med. 2021 Feb 12;11(2):120. doi: 10.3390/jpm11020120 (PMC7917624; doi:10.3390/jpm11020120)
Supplement: Supplementary file 1 [file jpm-11-00120-s001.zip › Supplementary Table 2.docx]

| Table S2. Pooled proportion of patients with metachronous ACRN according to the index colonoscopy finding and age group | | |
| --- | --- | --- |
| Index colonoscopy finding | Age group | Pooled proportion, % (95% CI) |
| Any adenoma | ≥50 years | 8.1 (5.4-12.1) |
|  | <50 years | 5.0 (2.8-8.6) |
| LRA | ≥50 years | 5.4 (3.2-8.8) |
|  | <50 years | 4.6 (2.0-10.3) |
| HRA | ≥50 years | 10.6 (8.5-13.1) |
|  | <50 years | 7.3 (5.3-10.0) |
| ACRN, advanced colorectal neoplasia; LRA, low-risk adenoma; HRA, high-risk adenoma; CI, confidence interval | | |
