# Supplementary figures and images for: Comparison of Risk of Metachronous Advanced Colorectal Neoplasia in Patients with Sporadic Adenomas Aged < 50 Versus ≥ 50 years: A Systematic Review and Meta-Analysis
Source: J Pers Med. 2021 Feb 12;11(2):120. doi: 10.3390/jpm11020120 (PMC7917624; doi:10.3390/jpm11020120)

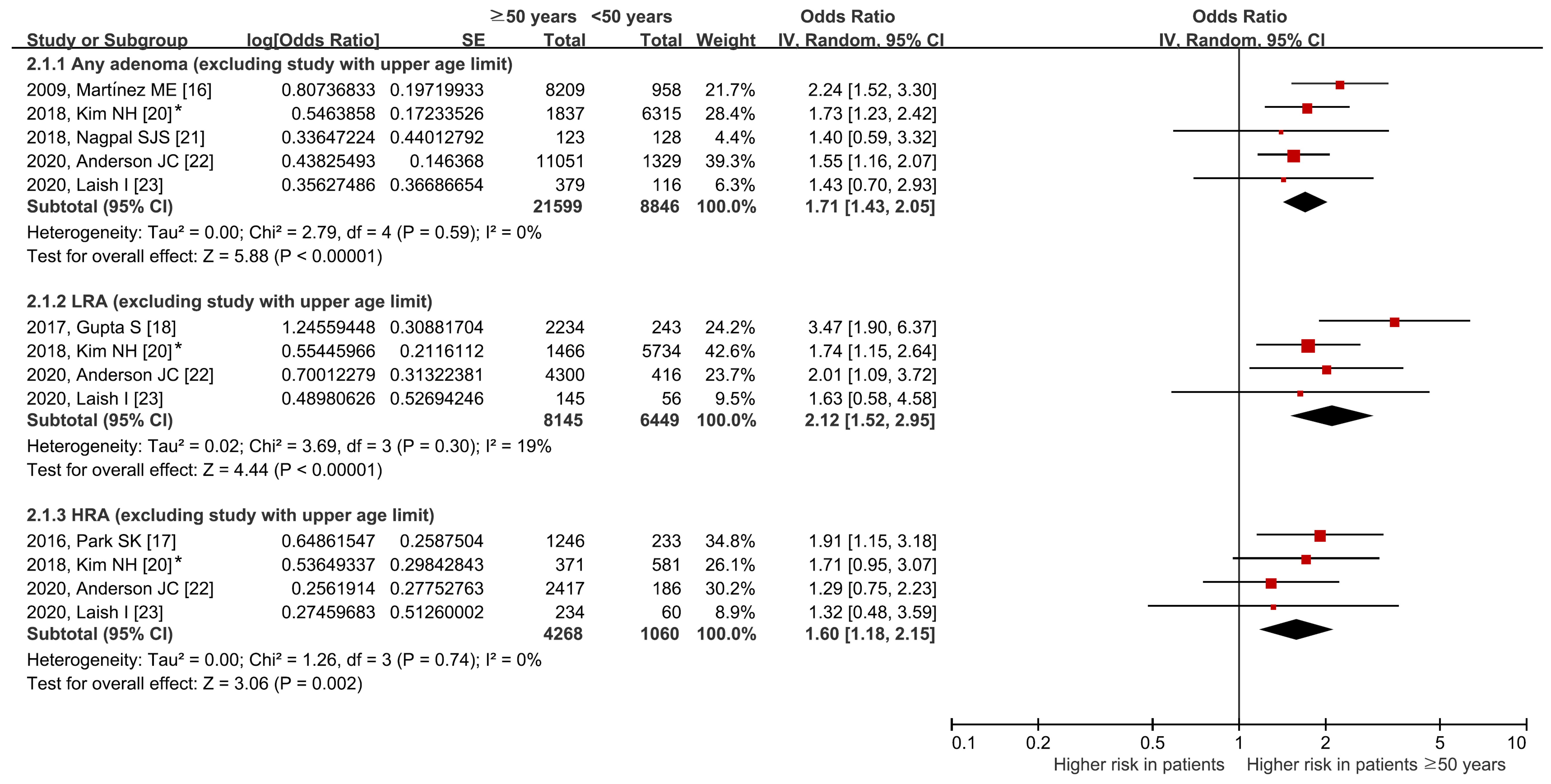

Supplement: Supplementary file 1 [file jpm-11-00120-s001.zip › Supplementary Figure 1.tif]
